# Machine learning‑based prediction of survival prognosis in esophageal squamous cell carcinoma

**DOI:** 10.1038/s41598-023-40780-8

**Published:** 2023-08-19

**Authors:** Kaijiong Zhang, Bo Ye, Lichun Wu, Sujiao Ni, Yang Li, Qifeng Wang, Peng Zhang, Dongsheng Wang

**Affiliations:** 1 Department of Clinical Laboratory, Sichuan Clinical Research Center for Cancer, Sichuan Cancer Hospital & Institute, Sichuan Cancer Center, Affiliated Cancer Hospital of University of Electronic Science and Technology of China, Chengdu, China; 2 Department of Radiation Oncology, Sichuan Clinical Research Center for Cancer, Sichuan Cancer Hospital & Institute, Sichuan Cancer Center, Affiliated Cancer Hospital of University of Electronic Science and Technology of China, Chengdu, China; 3 Department of Oncology, Tongji Hospital, Tongji Medical College, Huazhong University of Science and Technology, Wuhan, China

**Keywords:** Biomarkers, Risk factors, Cancer, Cancer models, Tumour biomarkers, Medical research, Biomarkers

## Abstract

The current prognostic tools for esophageal squamous cell carcinoma (ESCC) lack the necessary accuracy to facilitate individualized patient management strategies. To address this issue, this study was conducted to develop a machine learning (ML) prediction model for ESCC patients' survival management. Six ML approaches, including Rpart, Elastic Net, GBM, Random Forest, GLMboost, and the machine learning-extended CoxPH method, were employed to develop risk prediction models. The model was trained on a dataset of 1954 ESCC patients with 27 clinical features and validated on a dataset of 487 ESCC patients. The discriminative performance of the models was assessed using the concordance index (C-index). The best performing model was used for risk stratification and clinical evaluation. The study found that N stage, T stage, surgical margin, tumor grade, tumor length, sex, MPV, AST, FIB, and Mg are the important feature for ESCC patients’ survival. The machine learning-extended CoxPH model, Elastic Net, and Random Forest had similar performance in predicting the mortality risk of ESCC patients, and outperformed GBM, GLMboost, and Rpart. The risk scores derived from the CoxPH model effectively stratified ESCC patients into low-, intermediate-, and high-risk groups with distinctly different 3-year overall survival (OS) probabilities of 80.8%, 58.2%, and 29.5%, respectively. This risk stratification was also observed in the validation cohort. Furthermore, the risk model demonstrated greater discriminative ability and net benefit than the AJCC8th stage, suggesting its potential as a prognostic tool for predicting survival events and guiding clinical decision-making. The classical algorithm of the CoxPH method was also found to be sufficiently good for interpretive studies.

## Introduction

Esophageal cancer (EC) is one of the most lethal malignancies worldwide with an extremely aggressive nature and low survival rate. According to global cancer statistics, there were an estimated 572,000 new cases and 509,000 deaths in 2018^1^. In China, esophageal squamous cell carcinoma (ESCC) is the predominant histological type, accounting for approximately 90% of cases. ESCC is characterized by rapid progression and poor prognosis^2,3^, with a 5-year survival rate of only 15.3% in advanced stages^4^. Despite advances in surgical techniques and the incorporation of multimodal therapies in recent years, the prognosis of ESCC remains unsatisfactory^5^. Certain biomarkers for the prediction of ESCC prognosis could play a fundamental role in the clinical management of each patient and have important implications regarding the choice of optimal medical therapy for secondary prevention^6–9^. However, effective tools for clinical daily work are currently lacking. Therefore, there is an urgent need to identify novel prognostic biomarkers or develop an integrated prediction model for clinical prediction.

Clinical prediction models that integrate clinicopathological parameters, laboratory indexes, and survival outcomes using big data from large cohorts of patients have the potential to guide clinical decision-making and therapeutic prognoses^10–12^. Despite significant efforts to explore the prognosis of ESCC, current prognostic models remain imperfect^13–16^. Previous studies have mainly focused on the prognostic evaluation of a small number of clinical indicators using univariate and multivariate analysis^14–17^. Furthermore, most ESCC prediction models have been developed using traditional statistical approaches such as CoxPH regression or logistic regression, without proper evaluation mechanisms to determine the best performing model prior to model building^13–17^. Additionally, the sample sizes and assessed predictors in these studies are often limited, leading to poor reproducibility of model performance and insufficient evidence for clinical applications^14–17^. Therefore, there is a need to develop more comprehensive and reproducible prediction models for ESCC that can be effectively used in clinical practice.

The emergence of machine learning has presented a potential solution to the issue of poor reproducibility in the development of clinical prediction models based on complex clinical information^18^. Machine learning is an interdisciplinary field that combines computer science and computational statistics to improve the efficiency of disease prognosis and therapeutic decision-making. Machine learning approaches can overcome some of the limitations of current analytical methods by utilizing computer algorithms to handle multi-dimensional variables, identify non-linear relationships between clinicopathological features and outcomes, and develop accurate prediction models more efficiently^11,19^. Machine learning-based algorithms have been widely applied in medical science, particularly in predicting cancer diagnosis and prognosis^18^. For example, Abuhelwa et al.^10^ developed a machine learning model for survival prediction in urothelial cancer (UC) patients treated with atezolizumab, which found that the GBM model outperformed other models such as CoxBoost, random forest, and GLM in predicting patients' survival. D'Ascenzo et al.^11^ also developed a PRAISE score based on four machine learning models for the prediction of 1-year post-discharge all-cause death, myocardial infarction, and major bleeding.

Developing an accurate prediction model is crucial for guiding clinical decision-making, and the key to achieving this is to identify the best-performing algorithm. To date, no studies have employed machine learning algorithms with laboratory indicators to predict prognosis in ESCC patients. Therefore, this study aims to develop a prognostic model using six different machine learning approaches, which could potentially be used to facilitate individualized patient management strategies.

## Methods

### Study cohort

The objective of this study was to investigate consecutive cases of newly diagnosed ESCC patients who underwent esophagus surgery at Sichuan Cancer Hospital between January 2009 and December 2017. The inclusion criteria were as follows: (1) post-histologically confirmed ESCC without distant metastasis, (2) non-cervical esophageal cancer, (3) without previous anticancer therapy, and (4) complete clinical, blood parameters, and follow-up data. The exclusion criteria were as follows: (1) with a history of other malignancies or perioperative mortality, (2) the neck was invaded with cancer, (3) follow-up information was incomplete, and (4) follow-up shorter than 6 months.

A total of 2441 ESCC patients were enrolled in the study and randomly divided into two datasets. The training cohort (80%) was utilized for model development and parameter tuning, while the testing cohort (20%) was employed for model validation. All patients included in the study were staged according to the American Joint Committee on Cancer (AJCC) 8th edition TNM classification system.

### Predictors and outcomes

Among eligible cases, 27 predictors included patient clinicopathological characteristics, laboratory indicators, and survival outcomes that were prospectively collected from medical records. (1) clinicopathological characteristics: age, sex, Karnofsky performance scale (KPS) score, tumor length, tumor grade, tumor location, vascular invasion, surgical margin, dissected lymph nodes (LN) number, nerve invasion, T stage, N stage, AJCC8th stage, treatment. The primary treatment options include surgical intervention alone, followed by adjuvant chemotherapy (CT), radiotherapy (RT), and concurrent chemoradiotherapy (CCRT) after surgery. The surgical methods for esophageal cancer include endoscope (thoracoscopy or laparoscopy) surgery, and thoracotomy surgery. The synchronous chemotherapy regimens for esophageal cancer typically include monotherapy with platinum-based agents, monotherapy with fluorouracil, combination of paclitaxel with platinum agents, combination of cisplatin with fluorouracil or capecitabine, combination of paclitaxel with fluorouracil or capecitabine, and combination of oxaliplatin with fluorouracil or capecitabine. (2) laboratory indicators: hematocrit (HCT), mean platelet volume (MPV), neutrophil-to-lymphocyte ratio (NLR), monocytes (MONO), eosinophils (EO), direct bilirubin (DBIL), albumin (ALB), aspartate aminotransferase (AST), alkaline phosphatase (ALP), sodium (Na), magnesium (Mg), fibrinogen (FIB), lymphocyte -to- monocytes ratio (LMR). The predicted outcome was overall survival (OS), which was defined as the time from the date of surgery to death or the last follow-up. The model’s predictive ability was assessed in 1, 3, and 5- years.

### Feature selection and importance

The LASSO regularization and univariable Cox regression analysis was used to perform variable filtering. The LASSO regularization could penalize the absolute values of some coefficients toward zero, so it will remove the less important features from the model. This method has proven to be useful for feature selection in problems with a large number of covariates. Variables with p-values less than 0.05 in univariable Cox analyses were used for subsequent model development. The ranked importance of each feature was calculated by using permutation importance, and the optimal features were extracted after tuning the model parameters with 10-fold cross-validation resampling using the sequential backward search method from the final model. If permuting the values of a feature reduces the discriminative power of the model, it is considered important because the model relies heavily on that feature to make predictions. The high-ranked features will be considered more relevant and the ones with low rank could be excluded.

### Model development and validation

Six machine learning algorithms including Recursive Partitioning and Regression Trees (Rpart), Elastic Net Regularized Generalized Linear Models (Elastic Net), Gradient boosted machine (GBM), random survival forest (randomForestSRC), Gradient Boosting with Component wise Linear Models (GLMboost), and machine learning techniques extended Cox proportional hazards (CoxPH) were utilized to fit models that predicted survival outcomes. Rpart is a classification, regression and survival trees algorithm based on recursive partition, which it generates a tree structure by recursive binary partition of the data set, and each leaf node represents a category or a numerical value. In the process of constructing the decision tree, Rpart considers several partition variables and points, as well as pruning, so that the generated model has better generalization ability and prediction ability^20^. Elastic-net regularization is a flexible solution between Ridge and Lasso, as it combines both L1 and L2 penalties under a parameter called alpha. This method provides the strength of both types of regularization, since the lasso optimizes feature selection and interpretability while Ridge allows grouping effect^21^. GBM is a decision tree based ensemble learning algorithm, which improves the prediction ability of the model by iteratively training a series of decision trees. GBM performs well on many machines learning tasks, including classification, regression, and survival^22^. Random Survival Forests is a machine learning algorithm used for survival analysis. It is an extension of the Random Forest algorithm and is used to predict the survival time of an individual based on a set of predictor variables. It has several advantages over other survival analysis methods. They are able to handle high-dimensional data and can capture complex non-linear relationships between the predictor variables and the survival outcome. They are also able to handle missing data and censoring, which is common in survival analysis^23^. GLMBoost is a gradient boosting tree based regression and classification algorithm that uses the generalized linear model (GLM) as the base model. GLMBoost uses a gradient boosting algorithm to progressively improve the predictive power of the underlying model while controlling the model complexity through regularization. One advantage of GLMBoost is that it can handle a wide range of data types, including categorical and continuous variables. It also has the ability to handle missing data, which is a common problem in real-world datasets^24^. Cox proportional hazards regression (CoxPH) is a method used in survival analysis to estimate the effect of a factor on survival time. The CoxPH model assumes that the proportional hazard is constant, i.e. the effect of a factor is constant over the entire observation period. CoxPH model can be used to analyze the incidence of illness, death, unemployment and other events.

Hyperparameter tuning for each model was conducted by using grid search with 5-fold cross-validation in the *mlr3tuning* package. The search space of hyperparameter was created by the *paradox* package. Each hyperparameter range was established and exhaustively adjusted to enhance the predictive performance of the models and ensure that they fit the data well. The specific hyperparameters for each model is shown Table S1. For the specific meaning of each parameter, please refer to the *rpart*, *gbm*, *glmnet*, *randomForestSRC* and *glmboost* packages. The model performance was evaluated by the learning metrics of the average concordance index(C-index) on the training set using grid search with 5-fold cross-validation repeated 20 times, and the best-performing model was selected for further study. The *mlr3*^25^ package was employed for model development and model implementation of machine learning.

The risk score of the final model was calculated to stratify patients into three risk groups (low, intermediate, and high) with thresholds reflecting clinically meaningful gradients in risks. Survival probabilities were assessed by using Kaplan-Meier curves with the R “*survminer*” package in different patient groups. The time receiver operating characteristic (ROC) curve, area under ROC curves (AUC) value, calibration curve, and decision curve analyses (DCA) were employed to access clinical use.

### Statistical analysis

The patient’s characteristics were described as number (%) for categorical variables and median (interquartile range [IQR]) or mean ± standard deviation (SD) for continuous variables, respectively. Categorical variables were compared using the Chi-square test or Fisher’s exact test when appropriate. The t-test was performed between parametric continuous variables, while the Mann-Whitney test or Kruskal-Wallis test was performed for non-parametric variables. All statistical analyses were performed using R software 4.1.3 (https://www.r-project.org/), and a two-sided p-value <0.05 was considered to indicate statistical significance.

### Ethical approval and consent to participate

This study was approved by the ethics committee of Sichuan Cancer Hospital (Grant No. SCCHEC-02-2020-015) and was conducted in accordance with the Guidelines for Good Clinical Practice and the Declaration of Helsinki. The informed consent requirement was waived by the ethics committee of Sichuan Cancer Hospital due to the retrospective design of the study.

## Results

### Clinicopathological characteristics

2441 ESCC patients were enrolled according to inclusion and exclusion criteria. 1954 patients were assigned to the training cohort and 487 patients were assigned to the validation cohort (Table 1). The median age of included patients was 62.0 years old (range, 34–90 years), and most patients were males (81.6%). The median follow-up time of OS was 28.23 months (range,6.10–115.3 months).

**Table 1:** Baseline features of included cohorts in different data sets.

Characteristics	All	Training cohort	Validation cohort
	*N* = *2441*	*N* = *1954*	*N* = *487*
Age	62.0 [57.0;67.0]	62.0 [57.0;67.0]	62.0 [57.0;67.0]
Treatment			
Surgery	1281 (52.5%)	1015 (51.9%)	266 (54.6%)
Surgery + RT	49 (2.01%)	45 (2.30%)	4 (0.82%)
Surgery + CT	834 (34.2%)	673 (34.4%)	161 (33.1%)
Surgery + CCRT	277 (11.3%)	221 (11.3%)	56 (11.5%)
Sex			
Male	1991 (81.6%)	1597 (81.7%)	394 (80.9%)
Female	450 (18.4%)	357 (18.3%)	93 (19.1%)
KPS score			
90–100	1393 (57.1%)	1110 (56.8%)	283 (58.1%)
70–80	1048 (42.9%)	844 (43.2%)	204 (41.9%)
Tumor length	4.00 [2.70;5.00]	4.00 [2.80;5.00]	4.00 [2.55;5.00]
Tumor grade			
Well differentiated	522 (21.4%)	404 (20.7%)	118 (24.2%)
Moderate differentiation	964 (39.5%)	782 (40.0%)	182 (37.4%)
Poorly differentiated	955 (39.1%)	768 (39.3%)	187 (38.4%)
Tumor location			
Lower chest	528 (21.6%)	426 (21.8%)	102 (20.9%)
Middle chest	1325 (54.3%)	1052 (53.8%)	273 (56.1%)
Upper chest	588 (24.1%)	476 (24.4%)	112 (23.0%)
Surgical margin			
R0	2330 (95.5%)	1860 (95.2%)	470 (96.5%)
R1	74 (3.03%)	61 (3.12%)	13 (2.67%)
R2	37 (1.52%)	33 (1.69%)	4 (0.82%)
Varscular invasion			
No	2020 (82.8%)	1616 (82.7%)	404 (83.0%)
Yes	421 (17.2%)	338 (17.3%)	83 (17.0%)
Nerve invasion			
No	1982 (81.2%)	1581 (80.9%)	401 (82.3%)
Yes	459 (18.8%)	373 (19.1%)	86 (17.7%)
Dissected LN number	20.0 [14.0;28.0]	20.0 [14.0;28.0]	19.0 [14.0;27.0]
T stage			
T1	307 (12.6%)	234 (12.0%)	73 (15.0%)
T2	483 (19.8%)	391 (20.0%)	92 (18.9%)
T3	1441 (59.0%)	1154 (59.1%)	287 (58.9%)
T4	210 (8.60%)	175 (8.96%)	35 (7.19%)
N stage			
N0	1115 (45.7%)	883 (45.2%)	232 (47.6%)
N1	716 (29.3%)	593 (30.3%)	123 (25.3%)
N2	407 (16.7%)	318 (16.3%)	89 (18.3%)
N3	203 (8.32%)	160 (8.19%)	43 (8.83%)
AJCC8th stage			
0-I	298 (12.2%)	227 (11.6%)	71 (14.6%)
II	803 (32.9%)	640 (32.8%)	163 (33.5%)
III	1063 (43.5%)	863 (44.2%)	200 (41.1%)
IV	277 (11.3%)	224 (11.5%)	53 (10.9%)
HCT	41.8 [38.9;44.6]	41.8 [39.0;44.6]	41.9 [38.9;44.5]
MPV	11.5 [10.3;12.7]	11.5 [10.3;12.7]	11.5 [10.3;12.6]
MONO	0.38 [0.29;0.48]	0.38 [0.29;0.48]	0.36 [0.30;0.46]
EO	0.13 [0.08;0.22]	0.13 [0.08;0.22]	0.13 [0.08;0.22]
DBIL	4.84 [3.61;6.39]	4.80 [3.64;6.30]	4.90 [3.60;6.50]
ALB	43.0 [40.4;45.2]	42.9 [40.4;45.2]	43.1 [40.7;45.2]
AST	23.0 [19.0;28.4]	23.0 [19.1;28.2]	22.9 [19.0;29.3]
ALP	88.3 [74.2;105]	88.0 [74.2;105]	89.7 [74.2;108]
Na	141 [140;142]	141 [139;142]	141 [140;142]
Mg	0.95 [0.88;1.01]	0.95 [0.88;1.01]	0.95 [0.88;1.01]
FIB	3.20 [2.67;3.81]	3.19 [2.67;3.81]	3.23 [2.67;3.82]
NLR	2.52 [1.85;3.48]	2.52 [1.86;3.48]	2.55 [1.81;3.50]
LMR	3.99 [2.99;5.27]	3.98 [3.00;5.27]	4.00 [2.97;5.24]

### Model development of machine learning

**Figure 1:**
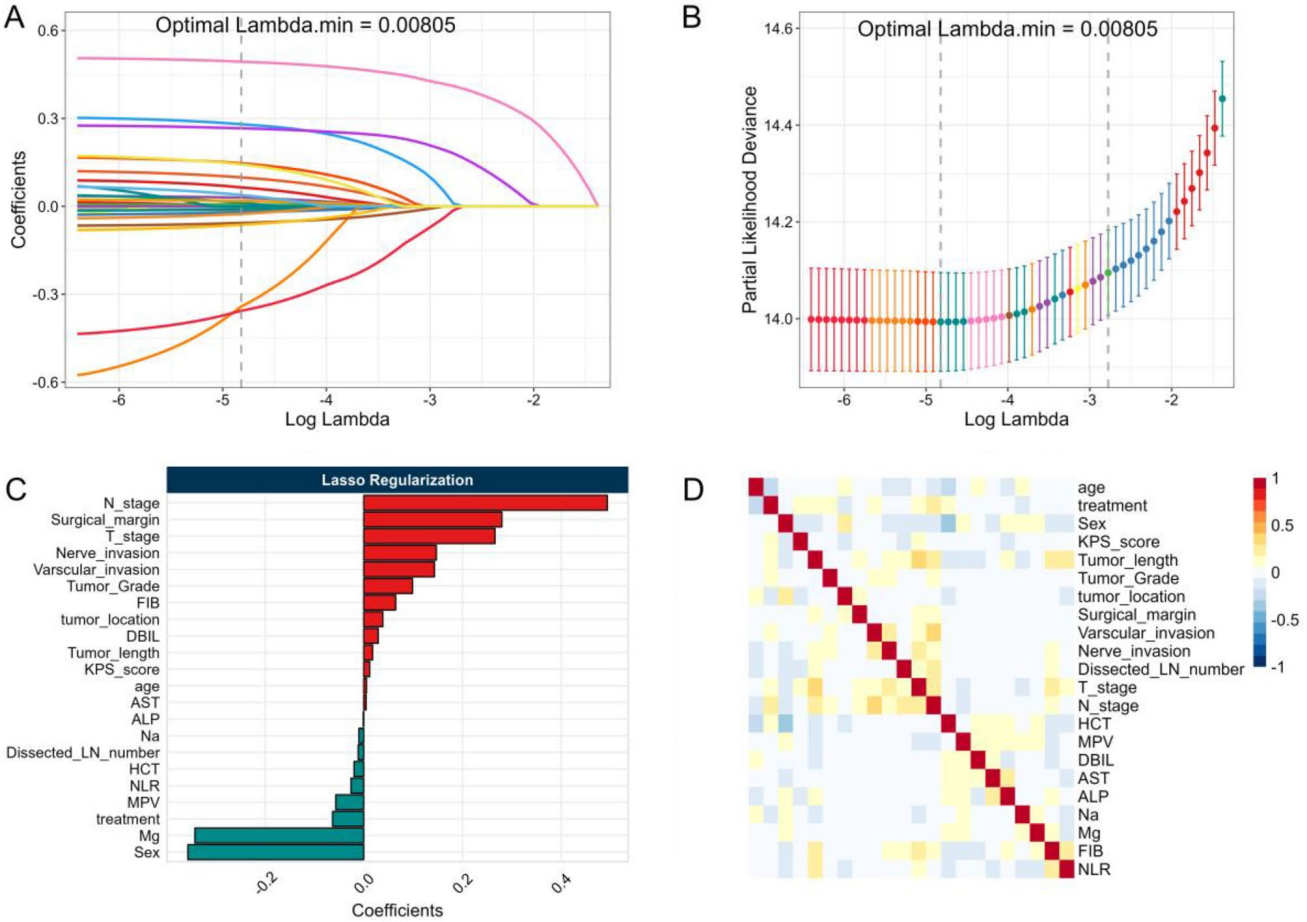
Feature selection of the patient's indicators by the LASSO regularization: (**A**) The relationship between LASSO penalty and regression coefficient change; (**B**) Cross-validation plot of partial-likelihood deviance curve with Log(λ) value in feature selection; (**C**) The coefficients of feature parameter estimation in the LASSO regularization; (**D**) Variable correlation plot of clinical features in the LASSO regression algorithm.

To prevent overfitting or uncertainty in the model, we first examined the correlation between continuous variables by spearman method before developing the model. We observed a slight collinearity problem between variables, as shown in Figure S1. We then utilized LASSO regression to penalize and select the optimal features, removing less important features from the model and reducing the correlation between variables. Ultimately, 22 variables were selected for model building with an optimal lambda.min of 0.00805, as shown in Fig. 1. Subsequent univariate COX regression analysis identified 14 significant factors for predicting patients' overall survival, including sex, KPS score, tumor length, tumor grade, surgical margin, vascular invasion, nerve invasion, T stage, N stage, MPV, AST, Na, Mg, and FIB (Table S2). Therefore, these 14 variables were selected for subsequent model development.

**Figure 2:**
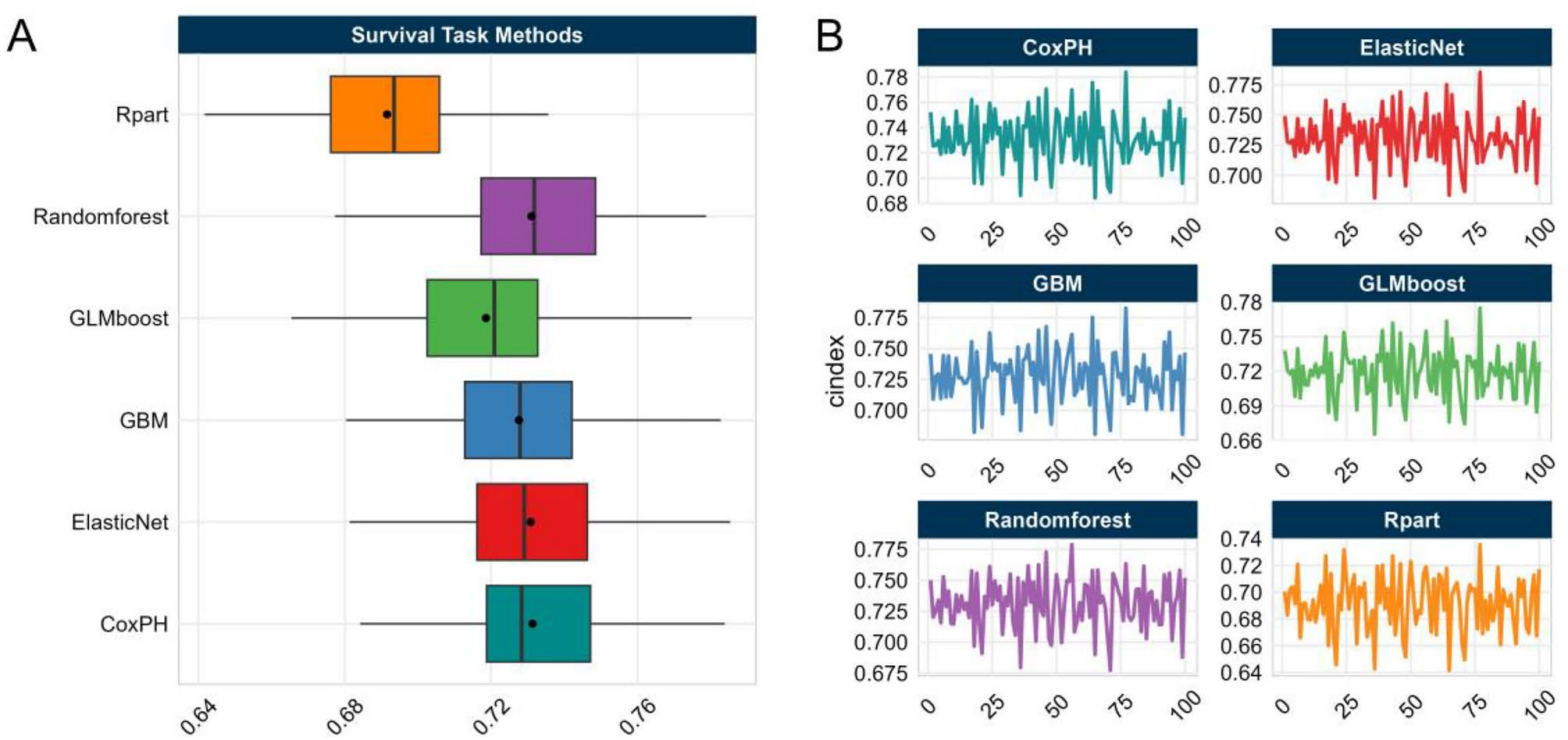
Prediction performance for the six-survival analyzing algorithm. (**A**) The c-index value was computed for each method using nested 5 × 20 cross-validations. (**B**) The confidence interval of the c-index value for each method using nested 5 × 20 cross-validations.

Six different survival analysis algorithms were utilized to model development in the training set. The hyperparametric search space and tuning results were given in Table S1. The discriminative performance of the developed models was evaluated by the average C-index using grid search with fivefold cross-validation repeated 20 times. The results were presented in Fig. 2 and Table 2, which demonstrate that the machine learning-extended CoxPH model, Elastic Net, and Random Forest exhibit similar performance in model cross-validation, with a C-index of 0.731. Furthermore, their prediction performance is superior to that of GBM, GLMboost, and Rpart. Considering the importance of model interpretability, we ultimately selected the classical algorithm of CoxPH regression as our final method for further study.

**Table 2:** Prediction performance of the machine learning methods.

Learners	C-index	SD	CV
CoxPH	0.731	0.021	0.028
Elastic Net	0.731	0.021	0.029
GBM	0.728	0.022	0.030
GLMboost	0.719	0.022	0.030
Random forest	0.731	0.021	0.029
Rpart	0.692	0.021	0.030

**Figure 3:**
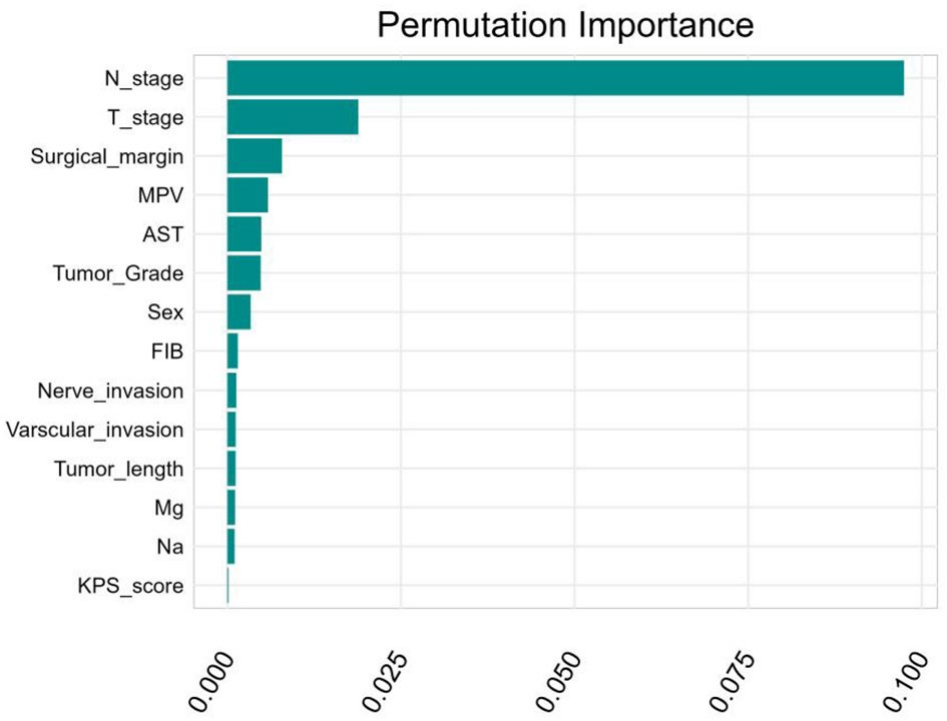
The ranked importance of the candidate variables.

Next, we utilized permutation importance method to calculate the ranked importance of 14 variables that were selected from the univariate Cox regression analysis, and the results are presented in Fig. 3. N stage, T stage, surgical margin, MPV, and AST were identified as the top 5 important predictors for predicting survival events. The optimal model features were extracted after tuning the model parameters with tenfold cross-validation resampling using the sequential backward search method. The final 10 features selected for CoxPH model building were N stage, T stage, surgical margin, MPV, AST, tumor grade, sex, FIB, tumor length, and Mg.

To estimate the impact of each predictor on mortality risk in the CoxPH model, we display the marginal effects of each factor in Figure S2. Our results demonstrate that T stages and N stages are significant risk factors in the CoxPH model, with the risk of mortality increasing with higher T and N stages. Females exhibit a lower risk of mortality than males. Positive surgical margins and poorly tumor grade increase the risk of mortality. Additionally, lower levels of MPV and Mg and higher levels of tumor length, AST, and FIB are associated with a greater risk of mortality in the model.

### Machine learning model performance

**Figure 4:**
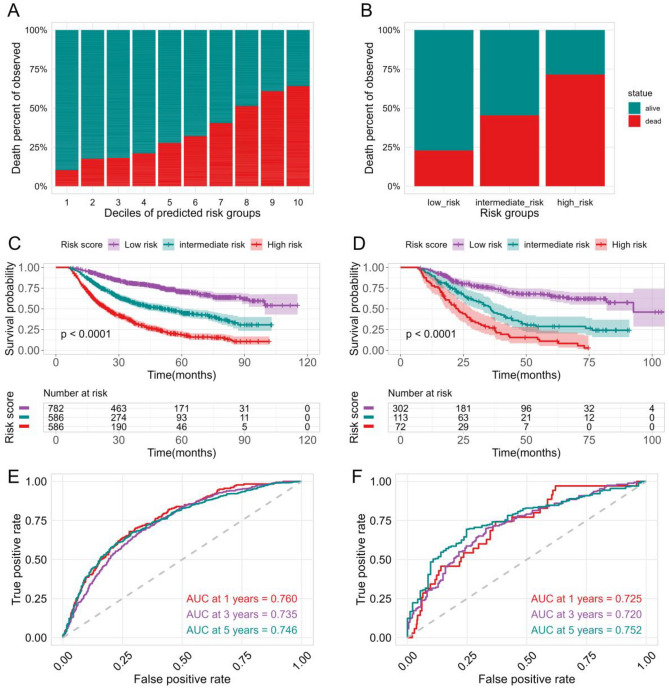
The survival prediction performance of machine learning-extended CoxPH model. (**A**) The percent of observed death according to deciles of event probability. (**B**) Three risk groups were stratified by similar patterns of survival distribution. Kaplan–Meier curves estimated the survival probabilities in the training (**C**) and validation (**D**) cohorts. Time ROC curves compared the performance of the risk mode at 1,3 and 5-year follow-up time in the training (**E**) and validation(**F**) cohorts.

With 10 prognostic features, patients were stratified into estimated risk deciles. We observed similar survival distributions for three risk scores and stratified the deciles of event probability into low, intermediate, and high-risk groups based on the related risks. The first to fourth deciles were classified as low-risk subgroups, with the percentage of observed death being significantly less than 25%. The eighth to tenth deciles were classified as high-risk subgroups, with the percentage of observed death exceeding 50%. The remaining groups were stratified into intermediate-risk groups (fifth to seventh deciles) (Fig. 4A,B).

Kaplan–Meier curve plots of survival probabilities revealed significant differences in survival rates among the high-, intermediate-, and low-risk subgroups in both the training and validation cohorts (Fig. 4C,D, all p < 0.0001). The risk stratification predicted 3-year overall survival probabilities of 80.8%, 58.2%, and 29.5% for low-, intermediate-, and high-risk subgroups, respectively, in the training cohort, and 75.4%, 48.8%, and 26.9% in the validation cohort. In addition, the risk stratification predicted 5-year overall survival probabilities of 70.6%, 45.6%, and 18.7% for low-, intermediate-, and high-risk subgroups, respectively, in the training cohort, and 65.3%, 27.9%, and 11.0% in the validation cohort (Table 3). The AUC values for 1-, 3-, and 5-year overall survival were 0.760, 0.735, and 0.746 in the training cohort, respectively, and a similar discriminative performance was observed in the validation cohort with AUC values of 0.725, 0.720, and 0.752 for 1-, 3-, and 5-year overall survival, respectively (Fig. 4E,F).

**Table 3:** 3,5-year OS survival probability of CoxPH model-based risk stratification in training and validation cohorts.

Risk groups	Training cohort		Validation cohort	
	3-year survival	5-year survival	3-year survival	5-year survival
Low risk	80.8% (77.9–84.0)	70.6% (66.5–74.9)	75.4% (70.3–80.9)	65.3% (59.0–72.2)
Intermediate risk	58.2% (53.9–62.7)	45.6% (40.8– 51.0)	48.8% (39.6–60.1)	29.7% (20.3–40.6)
High risk	29.5% (25.8–33.7)	18.7%(15.1–23.0)	26.9%(18.1–40.1)	11.0% (5.1–23.6)

**Figure 5:**
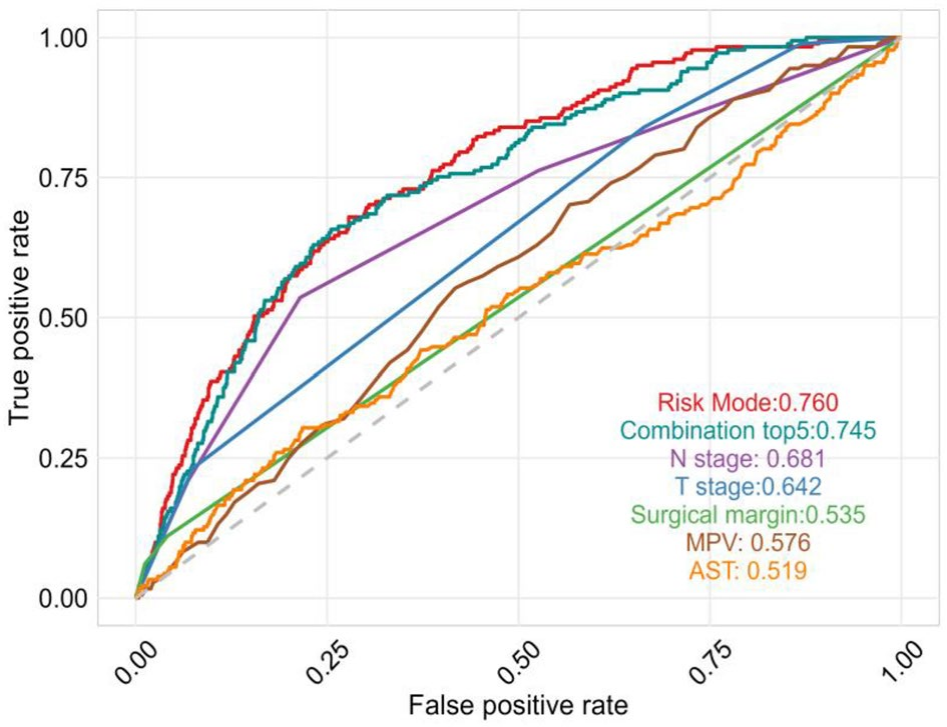
ROC curves to evaluate the capability of risk models and other indicators for ESCC patients' survival prediction.

We further evaluated the performance of the risk model by selecting the top 5 most important features (N stage, T stage, surgical margin, MPV, AST) from the permutation importance results for model development. Our findings demonstrate that the CoxPH risk model exhibits a significant advantage over the combination of these top 5 features, as well as individual features such as N stage (0.681), T stage (0.642), surgical margin (0.535), MPV (0.576), and AST (0.519) (Fig. 5).

### Machine learning model evaluation

**Figure 6:**
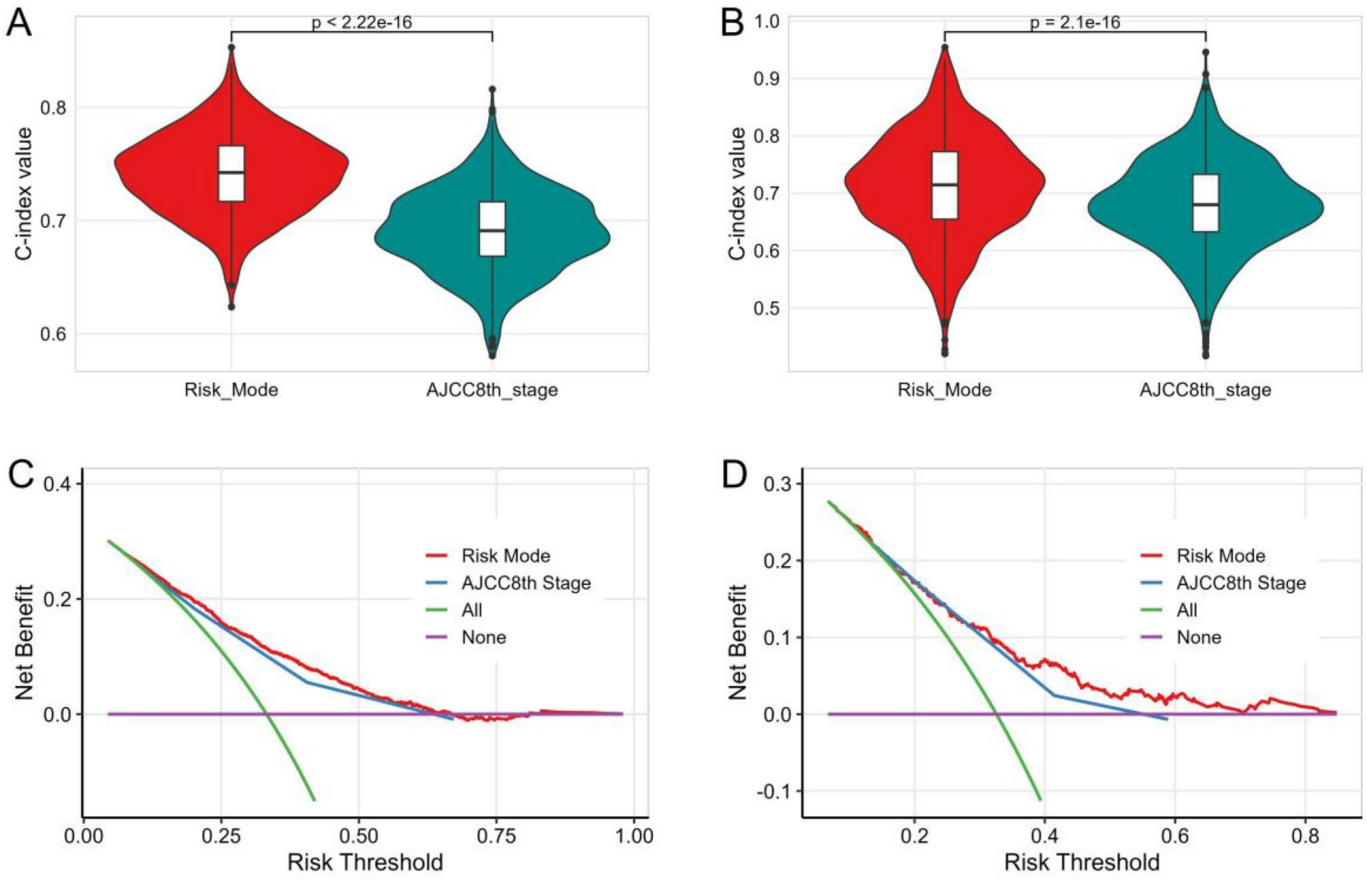
The C-index and decision curve analyses were performed to compare the performance between the risk score and the AJCC8th stage. The c-index values of the risk score and AJCC8th stage in training (**A**) and validation (**B**) cohorts by using fivefold cross-validation with 200 repeats; The net benefit of the risk model and AJCC8th stage in training (**C**) and validation (**D**) cohorts by using decision curve analyses.

**Figure 7:**
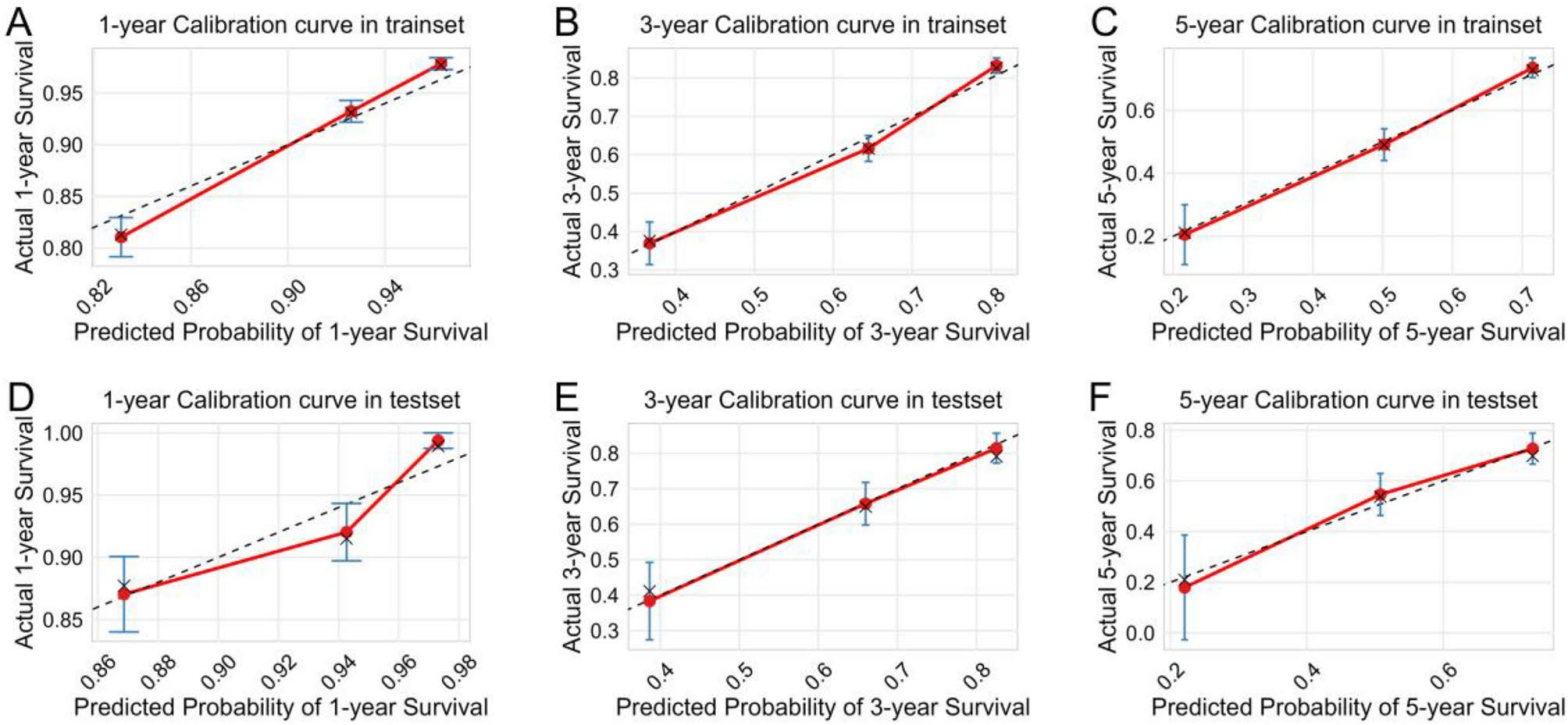
The calibration curve for predicting patient survival at 1 years (**A**), 3 years (**B**), and 5 years (**C**) in the training cohort and at 1 years (**D**), 3 years (**E**), and 5 years (**F**) in the validation cohort.

The machine learning-extended CoxPH risk model exhibits excellent predictive performance for survival events. However, it remains unclear whether the model can be utilized in clinical practice. Therefore, we compared the c-index values between the risk model and the AJCC8th stage using fivefold cross-validation with 200 repeats. Additionally, we employed calibration plots and DCA curves to evaluate the clinical utility of the model. Our results demonstrate that the risk model exhibits superior discriminative ability and net benefit over the AJCC8th stage for all patients in both the training and validation cohorts (Fig. 6). The calibration curve revealed a good agreement between predictions and actual observations for the probability of 1-, 3-, and 5-year survival (Fig. 7).

### The influence of treatment option on the model

In general, treatment options can impact the overall survival rate of patients. To clarify the impact of different treatment modalities on the overall survival of patients with ESCC, we evaluated the overall survival outcomes of different treatment subgroups among surgical intervention alone, CT, RT and CCRT treatment patients. However, we found no significant differences in the overall survival rates among the different treatment subgroups (Figure S3). In addition, we further evaluated the survival outcomes of ESCC patients who received surgical intervention alone, and found that the overall survival rate of ESCC patients who underwent endoscopic treatment was higher than those who underwent thoracotomy surgical resection (Figure S4). Furthermore, we also investigated the impact of chemotherapy on the overall survival of ESCC patients who underwent surgery, and found no significant differences in the overall survival rates among the different chemotherapy subgroups (Figure S5). These results suggest that ESCC patients who underwent endoscopic treatment may be in earlier stages of the tumor or have milder symptoms, while those requiring thoracotomy patients may be in advanced stages of the tumor. The patients who received thoracotomy may benefit from adjuvant radiotherapy or chemotherapy to improve their overall survival outcomes, achieving similar results as surgical intervention alone.

## Discussion

Machine learning approaches offer a technological innovation for personalized risk assessment^11^. In this study, we utilized high-quality clinical and laboratory data from a cohort of 2441 ESCC patients to develop and evaluate prediction models for ESCC patients' survival. Our findings indicate that the machine learning-extended CoxPH model demonstrated the best performance for predicting overall survival in ESCC patients. The risk scores derived from the CoxPH model effectively stratified ESCC patients into three prognostic risk groups with distinct survival events. These clinically meaningful risk scores exhibited excellent discriminative abilities, outperforming TNM AJCC8th stage in predicting patients' mortality risks. Accurately predicting mortality risks in ESCC patients remains an unmet need, and to our knowledge, this is the first study to compare the performance of different machine learning algorithms for developing and validating survival-prediction models in ESCC patients.

The use of machine learning to analyze big data offers significant advantages for assimilating and evaluating complex healthcare data^12^, and accurately forecasting cancer patients' survival is crucial for therapeutic decision-making and management^10,26,27^. While most machine learning-based models have been applied for cancer diagnosis and risk assessment, their application in survival prediction has been limited^28^. Furthermore, most machine learning-based survival analyses have been based on gene expression data from databases such as The Cancer Genome Atlas (TCGA)^18,29^ or multi-omics data^30^, with few studies utilizing high-dimensional real-world survival data^31,32^, thus limiting their applicability to the current practice. Recent research by Abuhelwa et al.^10^ demonstrated the feasibility and effectiveness of machine learning-based approaches for survival prediction in urothelial cancer patients treated with atezolizumab. In this study, we employed six machine learning algorithms to develop a prognosis model for 27 clinical variables in ESCC patients and found that the machine learning-extended CoxPH model, Elastic Net, and Random Forest have similar and excellent performance in predicting ESCC patients' survival and outperformed GBM, GLMboost, and Rpart models. Therefore, machine learning-based approaches for ESCC patients' survival prediction are feasible and effective, and the classical algorithms of the CoxPH method remain sufficiently good for interpretive studies.

Several indicators or scores have been developed to estimate the risk and management of ESCC patients based on research efforts investigating predictors of survival^13,15,16,33^. Previous studies have identified various factors associated with poor overall survival, including higher NLR and C-reactive protein-to-albumin ratio (CAR), perineural invasion, pathological stage, incomplete resection, neoadjuvant therapy^33,34^. We also confirmed that low preoperative serum sodium^15^ and low MPV^35^ were important risk factors for overall survival in ESCC patients, and the coagulation index which established PLT, MPV, and FIB could stratify patients into three risk groups with the 3-year OS rates for the low-, middle- and high-risk groups were 63.5%, 55.5%, and 43.1%, respectively^13^. In this study, we identified N stage, T stage, surgical margin, MPV, AST, tumor grade, sex, FIB, tumor length, and Mg as the most important features for predicting survival events. Higher T and N stages, positive surgical margins, poorly tumor grade were associated with increased mortality risk, while females have lower risk of mortality than males. Additionally, lower levels of MPV, Mg, and higher levels of tumor length, AST, FIB were also associated with a greater risk of mortality. Monitoring these clinical routine indicators can help predict prognostic risk and assist in clinical management strategies for ESCC patients. However, some previous findings may be biased due to small sample sizes or different methodologies^36^. Nevertheless, CoxPH risk scores derived from machine learning processes and large contemporary patient cohorts have the potential to overcome the shortcomings of existing predictors.

This study has several limitations that should be acknowledged. Firstly, it is an observational retrospective study, and the population included in the study is primarily concentrated in the Asian population, which could potentially introduce selection bias in model building. Additionally, the endpoint of our study was overall survival, and the prediction value for progression-free survival or disease-free survival remains unknown. Therefore, the efficiency of this model requires further systematic validation on larger cohorts by multicenter studies. In conclusion, we have developed and validated a machine learning risk model that can serve as a prognosis tool for predicting the survival of ESCC patients. Furthermore, the classical algorithms of CoxPH method remain sufficiently good for interpretive studies, and machine learning-based approaches are feasible for enhancing the optimization of disease prognosis and clinical decision-making.

### Supplementary Information

Supplementary Information.

## Data Availability

The authors declare that all data generated or analyzed for this study are available within the paper and its supplementary information. Additional raw data are available from the corresponding author upon reasonable request.
