# Supplementary material for: Machine learning‑based prediction of survival prognosis in esophageal squamous cell carcinoma
Source: Sci Rep. 2023 Aug 19;13:13532. doi: 10.1038/s41598-023-40780-8 (PMC10439907; doi:10.1038/s41598-023-40780-8)
Supplement: Supplementary file 1 — Supplementary Information. [file 41598_2023_40780_MOESM1_ESM.docx]

**Table S1.** Hyperparametric search space and results

| **Parameters** | **Range** | **Result** |
| --- | --- | --- |
| Coxph | none | none |
| GLMboost |  |  |
| mstop | [50,500] | 400 |
| nu | [0.1,1.0] | 0.5 |
| GBM |  |  |
| n.trees | [100,1000] | 100 |
| shrinkage | [0.001,0.1] | 0.034 |
| interaction.depth | [1,5] | 3 |
| RandomForest |  |  |
| ntree | [100,1000] | 600 |
| mtry | [1,10] | 2 |
| nodesize | [1,20] | 14 |
| Elastic Net |  |  |
| alpha | [0,1] | 0 |
| lambda | [0.001,0.1] | 0.067 |
| Rpart |  |  |
| cp | [0.001,0.1] | 0.001 |
| minsplit | [1,20] | 20 |

**Table S2.** Univariate Cox regression analysis

| **characteristics** | **HR** | **95% CI** | **pvalue** |  |
| --- | --- | --- | --- | --- |
| age | 1.008 | 1-1.016 | .061 | . |
| treatment | 1.030 | 0.972-1.092 | .318 |  |
| Sex | 0.581 | 0.477-0.707 | < .001 | *** |
| KPS_score | 1.170 | 1.022-1.339 | .023 | * |
| Tumor_length | 1.112 | 1.079-1.147 | < .001 | *** |
| Tumor_Grade | 1.261 | 1.153-1.38 | < .001 | *** |
| tumor_location | 1.018 | 0.924-1.121 | .723 |  |
| Surgical_margin | 1.794 | 1.498-2.149 | < .001 | *** |
| Varscular_invasion | 1.784 | 1.518-2.096 | < .001 | *** |
| Nerve_invasion | 1.626 | 1.387-1.907 | < .001 | *** |
| Dissected_LN_number | 1.001 | 0.995-1.007 | .764 |  |
| T_stage | 1.699 | 1.544-1.869 | < .001 | *** |
| N_stage | 1.776 | 1.667-1.892 | < .001 | *** |
| HCT | 0.992 | 0.977-1.007 | .287 |  |
| MPV | 0.897 | 0.861-0.935 | < .001 | *** |
| DBIL | 1.022 | 0.993-1.051 | .140 |  |
| AST | 1.005 | 1.001-1.009 | .012 | * |
| ALP | 0.998 | 0.996-1.001 | .182 |  |
| Na | 0.949 | 0.924-0.974 | < .001 | *** |
| Mg | 0.438 | 0.221-0.867 | .018 | * |
| FIB | 1.210 | 1.122-1.304 | < .001 | *** |
| NLR | 1.007 | 0.976-1.039 | .657 |  |


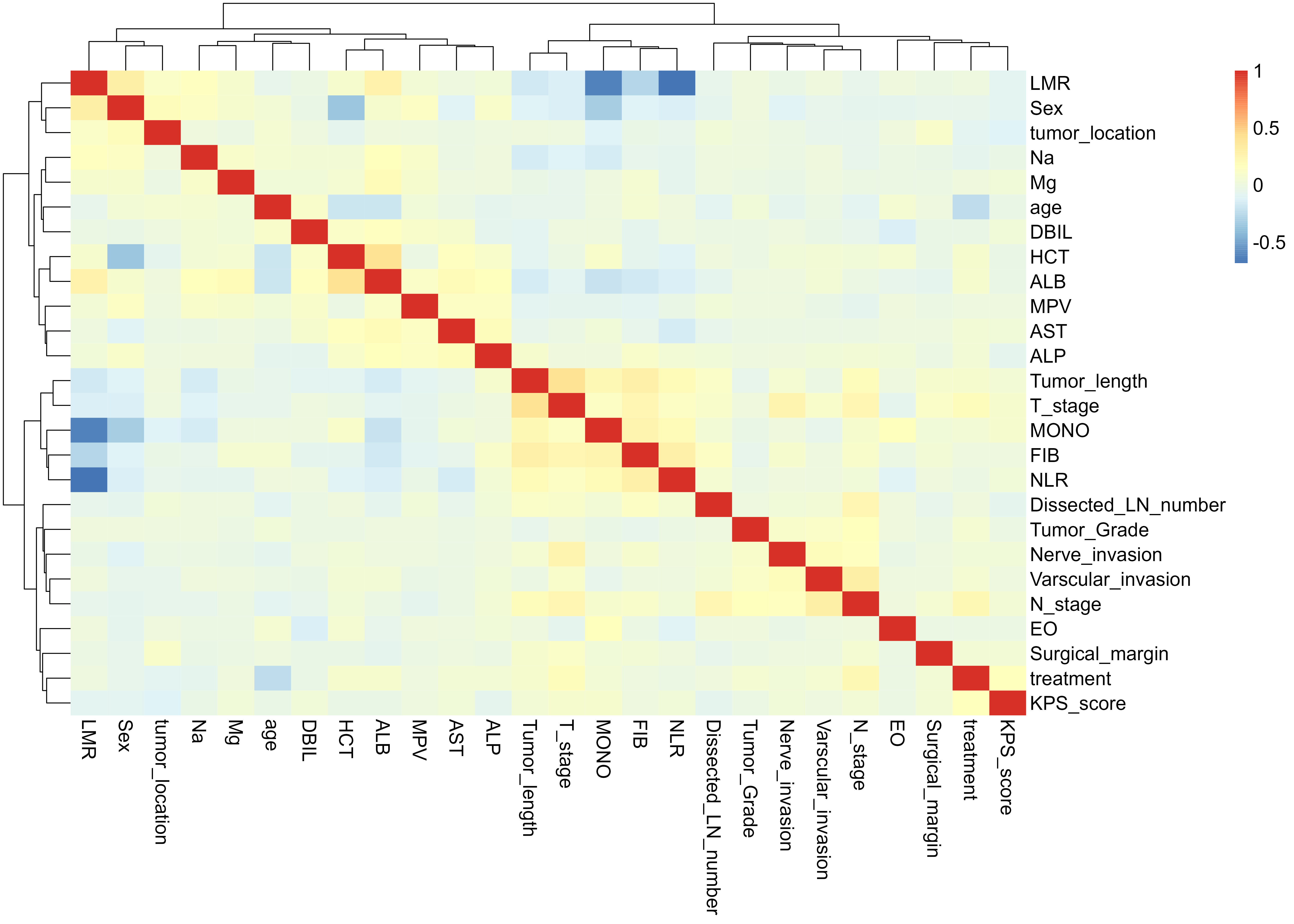


Figure S1. The variable correlation of laboratory indicators


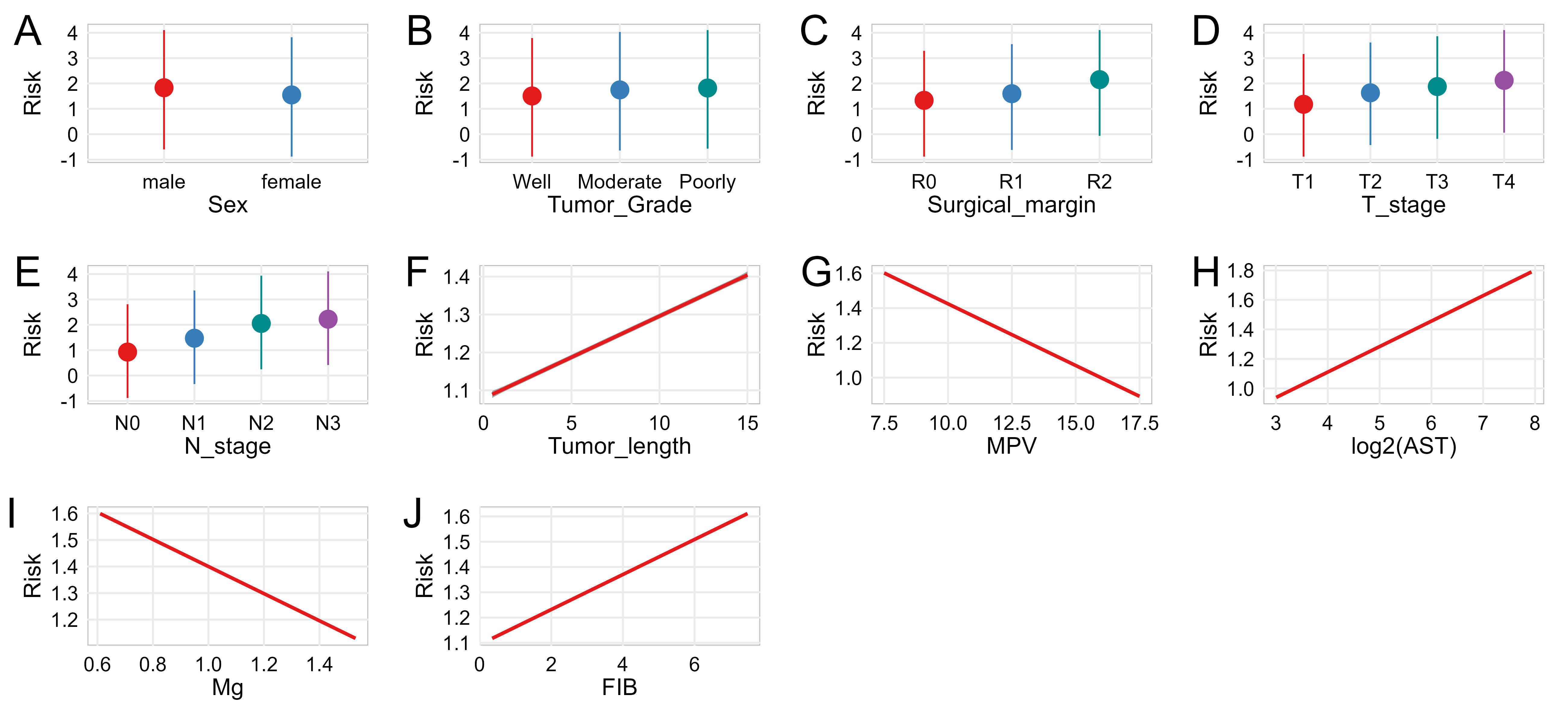


Figure S2. The marginal effect of risk factors in CoxPH model


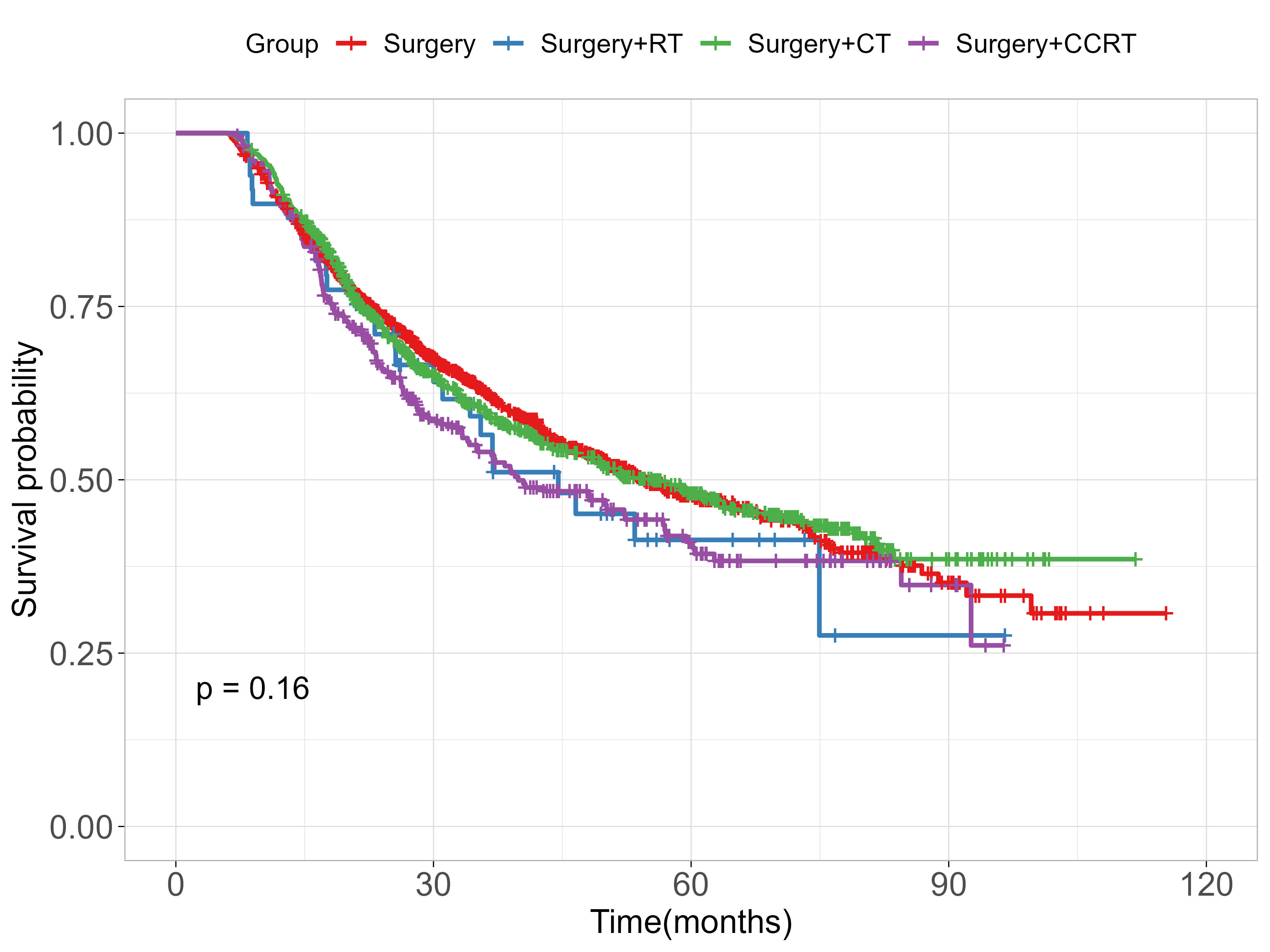


Figure S3. The survival probability among surgical alone, CT, RT and CCRT subgroups in ESCC patients.


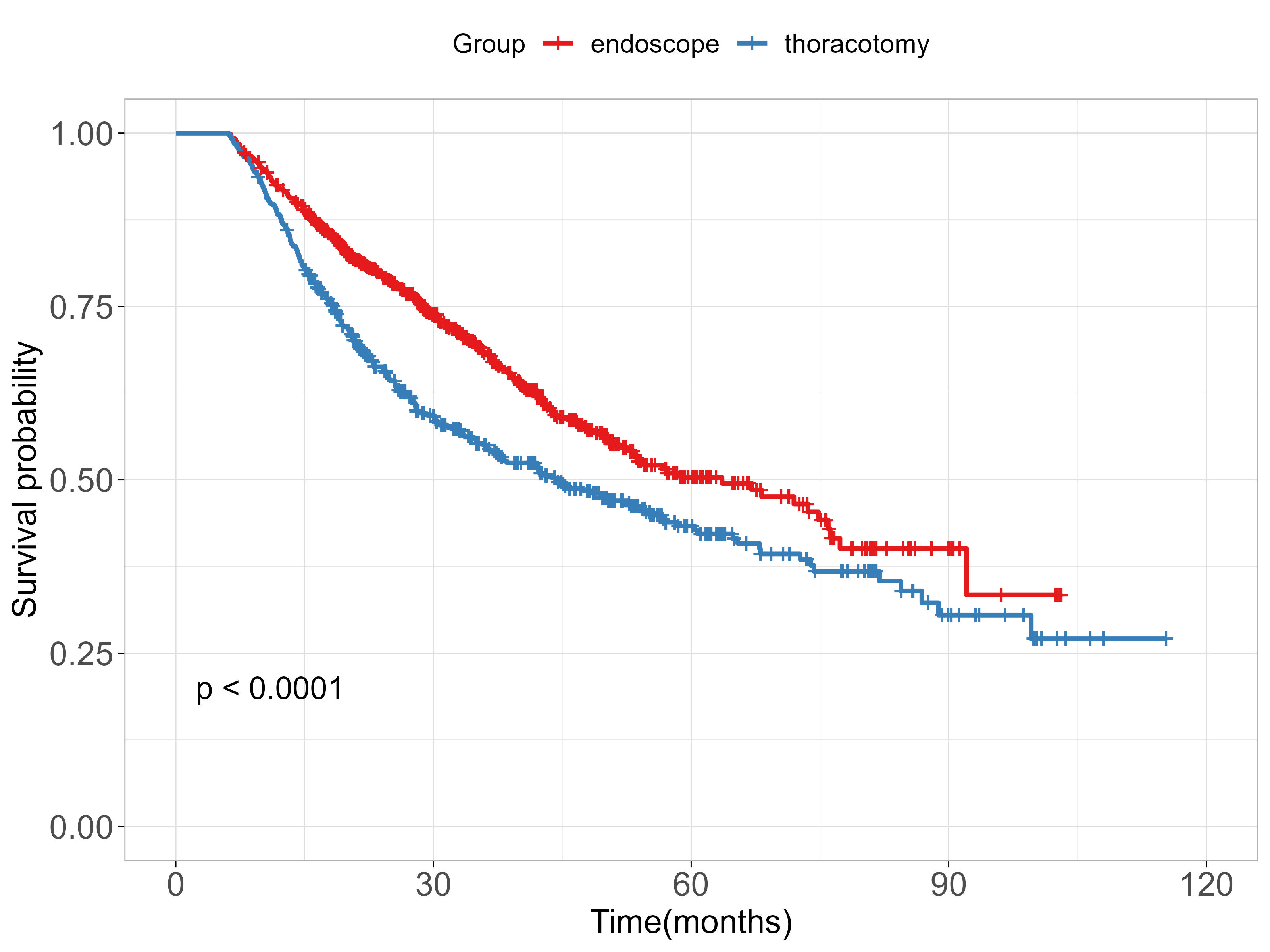


Figure S4. The survival probability between endoscopic surgery and thoracotomy subgroups in ESCC patients.


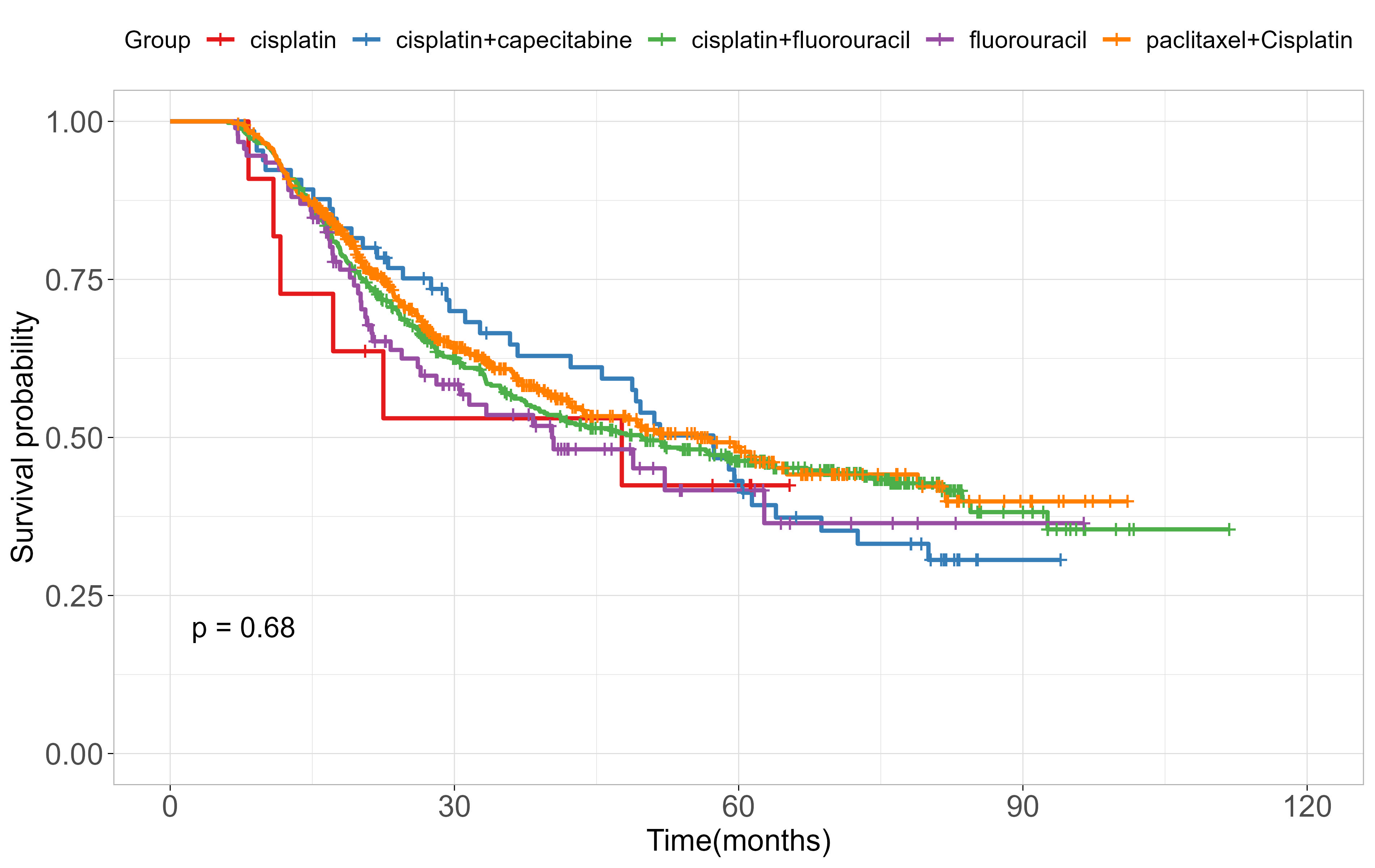


Figure S5. The survival probability of chemotherapy subgroups.
